# Identification of a quantitative trait loci (QTL) associated with ammonia tolerance in the Pacific white shrimp (*Litopenaeus vannamei*)

**DOI:** 10.1186/s12864-020-07254-x

**Published:** 2020-12-02

**Authors:** Digang Zeng, Chunling Yang, Qiangyong Li, Weilin Zhu, Xiuli Chen, Min Peng, Xiaohan Chen, Yong Lin, Huanling Wang, Hong Liu, Jingzhen Liang, Qingyun Liu, Yongzhen Zhao

**Affiliations:** 1 Guangxi Key Laboratory of Aquatic Genetic Breeding and Healthy Aquaculture, Guangxi Academy of Fishery Sciences, Nanning, 530021 China; 2 Key Lab of Freshwater Animal Breeding, Key Laboratory of Agricultural Animal Genetics, Breeding and Reproduction, Ministry of Education, College of Fishery, Huazhong Agriculture University, Wuhan, 430070 China; 3 Life Science Research Institute, Guangxi University, Nanning, 530004 China

**Keywords:** Genetic map, QTL, Transcriptomic, Ammonia tolerance, *Litopenaeus vannamei*

## Abstract

**Background:**

Ammonia is one of the most common toxicological environment factors affecting shrimp health. Although ammonia tolerance in shrimp is closely related to successful industrial production, few genetic studies of this trait are available.

**Results:**

In this study, we constructed a high-density genetic map of the Pacific white shrimp (*Litopenaeus vannamei*) using specific length amplified fragment sequencing (SLAF-seq). The constructed genetic map contained 17,338 polymorphic markers spanning 44 linkage groups, with a total distance of 6360.12 centimorgans (cM) and an average distance of 0.37 cM. Using this genetic map, we identified a quantitative trait locus (QTL) that explained 7.41–8.46% of the phenotypic variance in *L. vannamei* survival time under acute ammonia stress. We then sequenced the transcriptomes of the most ammonia-tolerant and the most ammonia-sensitive individuals from each of four genetically distinct *L. vannamei* families. We found that 7546 genes were differentially expressed between the ammonia-tolerant and ammonia-sensitive individuals. Using QTL analysis and the transcriptomes, we identified one candidate gene (annotated as an ATP synthase g subunit) associated with ammonia tolerance.

**Conclusions:**

In this study, we constructed a high-density genetic map of *L. vannamei* and identified a QTL for ammonia tolerance. By combining QTL and transcriptome analyses, we identified a candidate gene associated with ammonia tolerance. Our work provides the basis for future genetic studies focused on molecular marker-assisted selective breeding.

**Supplementary Information:**

The online version contains supplementary material available at 10.1186/s12864-020-07254-x.

## Background

The Pacific white shrimp (*Litopenaeus vannamei*) is the most widely cultivated and highest-yielding crustacean species in the world [1]. *L. vannamei* tolerates a wide range of salinities, grows rapidly, is highly disease resistant, and can be farmed at high densities [2]. However, high-density shrimp cultivation often leads to water quality deterioration [3]. The toxicological factors associated with poor quality water often negatively affect shrimp [4]. One of the most common toxicological factors affecting shrimp health is ammonia nitrogen (ammonia-N) [5]. In aquaculture water, ammonia-N is mainly found as non-ionic ammonia (NH_3_) and ionic ammonia (NH_4_^+^); these compounds are usually in dynamic equilibrium [6]. As NH_3_ has no electric charge, it is highly fat-soluble and can easily penetrate organismal cell membranes, leading to toxic effects [7]. In aquatic organisms, NH_3_ affects membrane stability, as well as physiology, biochemistry, and growth; shrimp exposed to NH_3_ may exhibit dyspnea, lack of appetite, decreased disease resistance, and even death [8–11].

The maintenance of low aquatic ammonia-N concentrations is required for successful shrimp farming [12]. However, aquatic physical and chemical properties are complicated, and may be affected by various factors such as weather, the local environment, and the introduction of artificial feeds. Thus, new breeds of ammonia-tolerant shrimp may improve industrial production and reduce economic losses. Marker-assisted selection has proven to be a useful strategy for the development of new breeds with dramatically improved trait characteristics [13], and the first step towards developing a new shrimp breed is to identify genes or markers associated with the desired trait [14]. Several previous studies have focused on the genetic bases of ammonia tolerance in shrimp. For example, Lu et al. identified 12 single nucleotide polymorphisms (SNPs) associated with ammonia tolerance in *L. vannamei* using marker-trait correlation analyses [15]. At the same time, Lu et al. identified 202 proteins that were significantly differentially expressed between ammonia-tolerant and ammonia-sensitive *L. vannamei* families using a comparative proteome analysis based on isobaric tags for relative and absolute quantification (iTRAQ) [16]. In addition, Jie et al. identified several pathways and genes involved in ammonia tolerance in *L. vannamei* based on comparative transcriptomic and metabolomic analyses of ammonia-tolerant and ammonia-sensitive *L. vannamei* families [17]. Finally, several studies identified transcriptomic changes and differentially expressed genes in *L. vannamei* after ammonia stress [9, 18]. However, no studies have investigated the quantitative trait loci (QTL) associated with ammonia tolerance in shrimp.

QTL analysis effectively identifies molecular markers or candidate genes associated with economically important traits in plants and animals [19]. QTL analyses usually require high-density genetic linkage maps. To date, genetic linkage maps have primarily been constructed using high-throughput sequencing technologies, such as restriction site-related DNA sequencing (RAD-seq), genotyping sequencing (GBS), and specific length amplified fragment sequencing (SLAF-seq) [20]. In particular, SLAF-seq efficiently identifies and genotypes large-scale SNPs [20]. SLAF-seq has been applied to many plant species, including spinach [21], sesame [22], walnut [23] soybean [24], cucumber [25], wax gourd [26], cauliflower [27], white jute [28], and maize [29]. SLAF-seq has also been successfully applied to *L. vannamei* [30].

Therefore, SLAF-seq was used in the current study to construct a high-density genetic map of *L. vannamei*. Furthermore, QTL analysis of ammonia tolerance in *L. vannamei* was performed. Transcriptomic differences between ammonia-tolerant and ammonia-sensitive individuals across several *L. vannamei* families were compared to identify potential candidate genes coferring ammonia tolerance within QTLs.

## Methods

### Preparation of the mapping family

The *L. vannamei* used in experiments were obtained from the shrimp-breeding center at the Guangxi Academy of Fishery Sciences (Nanning, Guangxi, China). The *L. vannamei* family used for mapping was constructed using artificial insemination. In brief, a male shrimp from a family with a relatively high ammonia-tolerance (obtained via 10 consecutive generations of breeding) was mated with a female shrimp from a common family. The hatched offspring were reared for about 1 year. Then, a male and female shrimp were randomly selected from the year-old offspring and mated. The F1 progeny were used for mapping (LV-N).

### Measurement of ammonia tolerance

A total of 284 shrimp (average body weight: 20.78 g) were randomly selected from the LV-N family. Selected shrimp were transferred to a 2 m × 4 m × 1 m indoor pool and allowed to acclimate for 1 week. Aquatic conditions during the acclimation and experimental periods were kept constant: temperature of 27.0 ± 0.5 °C, pH of 8.1 ± 0.2, salinity of 30.2‰, and dissolved oxygen of 6–8 mg/L; culture water was kept aerated, and shrimp were fed formulated pellets (Zhengda Feed, China) daily at a ratio of 5% body weight. Following acclimation, an acute ammonia stress test was performed. The ammonia-N concentration used for the acute stress test was 345.94 mg/L, based on the results of a preliminary experiment. This was the concentration at which half of the experimental shrimp died in 72 h under stress. The ammonia-N concentration of the water in the experimental pool was controlled by adding NH_4_Cl stock solution (prepared by dissolving analytically pure NH_4_Cl in filtered seawater). The concentration of ammonia-N in the water was measured daily using standard methods [31]. To keep the ammonia-N concentration constant, NH_4_Cl stock solution was added if the ammonia-N concentration was < 345.94 mg/L, and seawater was added if the ammonia-N concentration was > 345.94 mg/L. During the experiment, shrimp heath was observed every hour, and dead shrimp were removed immediately. Shrimp were considered dead when lying motionless on the bottom of the pool and not responding to external stimuli. Collected dead shrimp were immediately frozen in liquid nitrogen and stored at − 20 °C for DNA extraction. The survival time of each shrimp was used as a proxy for ammonia tolerance. The experiment ended when all shrimp had died.

### DNA extraction

DNA was collected from the 284 F1 (LV-N) shrimp and the two parent shrimp. Marine animal genomic DNA extraction kits (Tiangen Biotech, China) were used to extract DNA from the tail muscle of each shrimp. DNA was quantified using a NanoDrop spectrophotometer and 1% agarose gel electrophoresis with a lambda DNA standard.

### SLAF library preparation and sequencing

First, we predicted the digestion of the *L. vannamei* genome (https://www.ncbi.nlm.nih.gov/genome/?term=Vannamei) [32] using self-developed software. We digested the extracted genomic DNA of all LV-N shrimp using the endonucleases identified by the predictive software. Then, dual-index sequencing adaptors were ligated to the DNA fragments obtained by digestion with T4 ligase, and the fragments were amplified using polymerase chain reactions (PCRs). PCR products (314–414 bp including the adaptor sequences) were purified and re-amplified using PCR. SLAF sequencing was carried out on an Illumina HiSeq system, following the Illumina-recommended procedure. To assess the accuracy of library construction, the same library-construction and sequencing steps using the genome of *Oryza sativa japonica* as a control was performed. Library construction and sequencing were performed by Biomarker Technologies Corporation (Beijing, China).

### SLAF-seq data analysis and genotyping

The raw sequencing reads were quality controlled by removing reads with a quality score < 20. The remaining raw reads were grouped by individual based on the dual-index adaptor sequences. The dual-index adaptor and 5-bp end sequences were then trimmed to obtain clean reads. The clean reads were mapped to the *L. vannamei* genome (https://www.ncbi.nlm.nih.gov/genome/?term=Vannamei) [32] using BWA [33]. Reads mapped to the same position with > 95% identity were considered the same SLAF. SNP-based polymorphic SLAF markers were identified by aligning reads from the same SLAF sequence. These polymorphic SLAF markers were then filtered by removing those with a parental sequencing depth less than 10-fold; those where the number of SNPs was > 5; those where the proportion of genotypes covering offspring was < 70%; and those with significant segregation distortion (chi-square test *P* < 0.05). The remaining polymorphic SLAFs were classified into eight separate patterns: aa × bb, ab × cd, cc × ab, ab × cc, ef × eg, hk × hk, nn × np, and lm × ll. Because the mapping population used in this study was an F1 population, the polymorphic SLAF with the pattern aa × bb was removed, and the remaining polymorphic SLAFs were used for the construction of the genetic map.

### Genetic map construction and QTL analysis

After coding the genotypes of the polymorphic SLAF markers, the genetic map was constructed using the single-chain clustering algorithm in HighMap [34], with the probability log threshold set to ≥5.0 and a maximum recombination rate of 0.4. The Kosanbi mapping function was used to convert percent recombination to genetic distance (cM). QTL analysis was conducted using the R/qtl software package [35]. The logarithm of odds (LOD) threshold was determined based on 1000 permutations (*P* < 0.05). The phenotypic variance explained by the QTL was estimated using the formula 1–10^–2LOD/*n*^, where n was the sample size [36].

### Transcriptome sequencing, candidate gene identification and quantitative real-time PCR (qRT-PCR) verification

To identify differentially expressed genes (DEGs) between ammonia-tolerant and ammonia-sensitive *L. vannamei*, the transcriptomes of 4 *L. vannamei* families were sequenced: the mapping family (LV-N) and three other randomly chosen common families (LV-A, LV-C, and LV-F) with different genetic backgrounds. Our previous analysis indicated that the 24-h median lethal concentration of NH4Cl was 140.96 mg/L, 189.19 mg/L, 117.88 mg/L, and 137.26 mg/L for families LV-A, LV-C, LV-F and LV-N, respectively (Supplementary Material, Table S1). Two hundred shrimp from each family were randomly selected, and subjected to the acute ammonia stress test (345.94 mg/L ammonia-N), as described above. In each family, 20 shrimp with the longest survival times (i.e., the most ammonia tolerant) were collected, as were the 20 shrimp with the shortest survival times (i.e., the most ammonia sensitive). When collecting the ammonia-sensitive shrimp, specimens that were out of balance and lying on the bottom of the pool were judged to be dying, and were collected immediately, without waiting for death. The hepatopancreas of each shrimp was extracted, and hepatopancreases were pooled to form an ammonia-tolerant sample and an ammonia-sensitive sample per family.

Total RNA was extracted from each pooled sample using TRIzol reagent (Invitrogen, USA), following the manufacturer’s instructions. Residual genomic DNA was removed with DNase I. RNA purity (OD260 / 280), concentration, and absorption peak were measured using a NanoDrop 2000. RNA integrity was assessed using an RNA Nano 6000 Assay Kit with an Agilent Bioanalyzer 2100. The isolated mRNA was divided into 100–400 bp fragments using an RNA fragment reagent (Illumina, USA). cDNA libraries were then constructed using NEBNext Ultra RNA Library Prep Kits (Illumina, USA), following manufacturer’s recommendations, and sequenced on an Illumina HiSeq system (Illumina, USA). Library construction and sequencing were performed by Biomarker Technologies Corporation (Beijing, China).

Raw sequencing reads were trimmed and filtered using in-house Perl scripts to remove adaptor sequences and low-quality reads; the Q20, Q30, GC-content, and sequence duplication levels of the clean data were calculated. Clean reads were then aligned to the *L. vannamei* genome (https://www.ncbi.nlm.nih.gov/genome/?term=Vannamei) [32] using Hisat2 2.1.0 (http://ccb.jhu.edu/software/hisat2/index.shtml) [37]. Matched reads were counted to determine gene expression levels using the fragments per kilobase of transcript per million mapped reads (FPKM) method [38]. DEGs were identified using edger [39]. unigenes were considered differentially expressed when the false discovery rate (FDR) was ≤0.01 and the fold change between groups was > 2. DEGs were functionally annotated against the following databases: Non-Redundant protein sequences (NR) (ftp://ftp.ncbi.nih.gov/blast/db/), Protein family (Pfam) (http://pfam.xfam.org/), Clusters of Orthologous Groups (http://www.ncbi.nlm.nih.gov/COG/), Swiss-Prot (http://www.uniprot.org/), Kyoto Encyclopedia of Genes and Genomes (KEGG) (http://www.genome.jp/kegg/), and Gene Ontology (GO) (http://www.geneontology.org/).

After obtaining DEGs, candidate genes among the DEGs were identified. We consider candidate genes associated with ammonia tolerance when (1) candidate genes located within the QTL interval; (2) candidate genes differentially expressed between the most ammonia-tolerant and the most ammonia-sensitive individuals in the mapping family (LV-N); (3) the regulation pattern (up- or down-regulated expression) of candidate genes between the most ammonia-tolerant and the most ammonia-sensitive individuals was consistent across the four families of *Litopenaeus vannamei* (LV-A, LV-C, LV-F, and LV-N).

qRT-PCR was used to validate the RNA-seq results by quantifying the expression of the candidate gene (LOC113809108) in the ammonia-tolerant and ammonia-sensitive pooled samples from the 4 *L. vannamei* families (LV-A, LV-C, LV-F, and LV-N). RNA-seq and qRT-PCRs analyses were carried out using the same samples. qRT-PCRs were performed using SYBR Premix Ex TaqTM II kits (TaKaRa, Japan), according to the manufacturer’s instructions. The primer sets used to detect LOC113809108 gene expression levels were designed using the Primer Premier software (version 5.0) [40] as follows: 5′-ACTTGGGTGCTGTAGCTCAA-3′ and 5′-CTCGACAGCAACCAGGGTAT-3′. *L. vannamei* 18S RNA was used as the internal reference gene; this gene was amplified using the primer sets as follows: 5′-GCCTGAGAAACGGCTACCACATC-3′ and 5′-GTAGTAGCGACGGGCGGTGTGT-3′ [41]. The qRT-PCR cycling program was as follows: preheating at 95 °C for 30 s, followed by 40 cycles of 95 °C for 5 s and 60 °C for 30 s. The qRT-PCR was carried out at 95 °C for 40 s, 95 °C for 5 s, and 62 °C for 30 s for 40 cycles. Three parallel qRT-PCRs were carried out for each sample. Relative gene expression levels were calculated using the 2-ΔΔCT method [42].

## Results

### Phenotypic variation

We developed an ammonia-tolerant shrimp family (designated LV-N) for mapping, and subjected 284 LV-N shrimp to an acute ammonia stress test. All shrimp died within 2–98 h, with a mean survival time of 65 h. Individual survival times were normally distributed and thus suitable for QTL detection. The accumulated mortality rate of shrimp is showed in Supplementary Material Fig. S1.

### SLAF-seq and genotyping

Based on the digestive enzyme prediction using the reference genome of *L. vannamei*, HaeIII and Hpy166II were used to digest the genomic DNA of the 284 LV-N shrimp for SLAF library construction. SLAF sequencing generated 439.77 gigabases (Gb) of data, consisting of 2201 megabases (Mb) of 100-bp paired-end reads. Across all reads, the average Q30 was 95.81%, the average GC content was 40.60%, and the GC distribution was normal (Table 1). The rice (*Oryza sativa japonica*) genome was used as a control to estimate the validity of the library construction. For the rice library, 343.21 Mb of data (1.72 Mb paired-end reads) were generated, with a Q30 of 95.81% and a GC content of 40.96%. In *L. vannamei*, 57.83% of the paired-end reads mapped successfully to the genome, as compared to 91.43% of the paired-end reads in rice. In addition, enzymatic digestion efficiency was 87.75% for *L. vannamei* and 92.19% for rice (Supplementary Material, Table S2). These results indicated that SLAF library construction and sequencing were adequate.

**Table 1:** Summary of the constructed genetic map of *Litopenaeus vannamei*

Map data	Value
Total bases	439.77 Gb
Total reads	2201.28 Mb
Average Q30	95.81%
Average GC	40.60%
Enzyme digestion protocol	HaeIII+Hpy166II
Restriction fragment length	314–414 bp
Percentage of reads matching the *L. vannamei* genome	57.83%
Average enzymatic digestion efficiency	87.75%
Predicted number of markers	339,517
Number of high-quality slafs	807,505
Number of polymorphic slafs	293,415
Number of SLAF markers on the map	17,338
Average depth in parents	208.90 ×
Average depth in offspring individual	38.55 ×
Number of linkage groups	44
Total distance of the map	6360.12 cM
Average distance of the map	0.37 cM

**Fig. 1:**
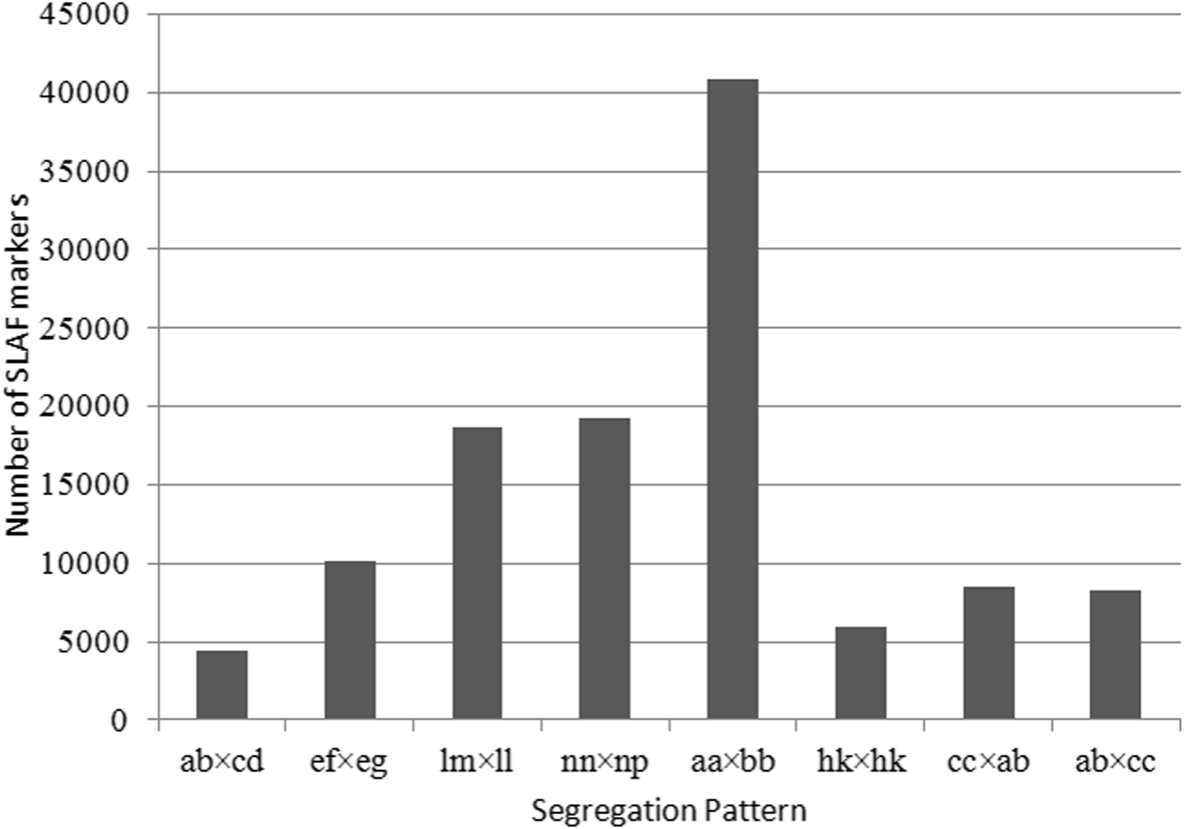
Number of markers associated with each of the eight polymorphic specific length amplified fragment (SLAF) segregation patterns

After filtering and clustering all reads, 807,505 SLAFs were identified. The average sequencing depth of these SLAFs was 42.8-fold for the male parent, 42.14-fold for the female parent, and 12.43-fold for the progeny (Table 1). Of the 807,505 high-quality SLAFs detected, 293,415 (36.34%) were polymorphic (Table 1). After further filtering, the remaining 115,973 SLAF markers were successfully classified into eight genotypic patterns: ab × cd, cc × ab, aa × bb, ab × cc, ef × eg, lm × ll, hk × hk, and nn × np. The most common pattern was aa × bb, followed by nn × np and lm × ll (Fig. 1). Because the mapped population was an F1 population, aa × bb were eliminated as a valid marker.

### Characteristics of the genetic map

**Fig. 2:**
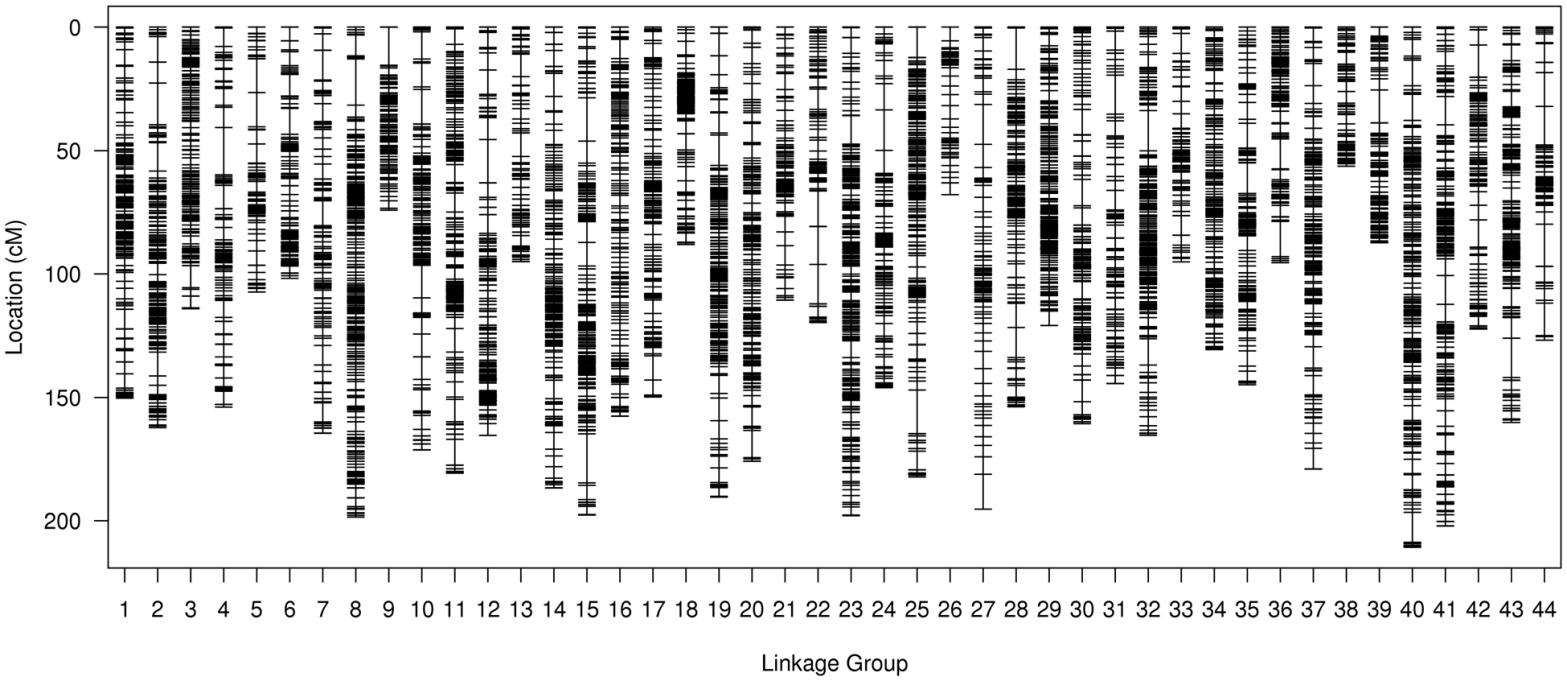
High-density linkage map of *Litopenaeus vannamei* showing genetic distances among specific length amplified fragment (SLAF) markers. Black bars represent SLAF markers

Linkage analysis labeled 17,338 SLAF markers on the genetic map: 11,512 on the male map, 10,293 on the female map, and 17,338 on the sex-average map (Fig. 2). Each map contained 44 linkage groups (LGs). The total distances on the male, female, and sex-average maps were 6604.99 cM, 5476.20 cM, and 6360.12 cM, respectively. The mean distance between adjacent markers was 0.58 cM on the male map, 0.53 cM on the female map, and 0.37 cM on the sex-average map (Supplementary Material, Table S3, Table S4, and Table S5). The distribution of markers among LGs was not uniform: in the male map, LG31 contained the most markers (585), while LG26 contained the least (39); in the female map, LG36 contained the most markers (540), while LG27 contained the least (21); and in the sex-average map, LG31 contained the most markers (695), while LG26 contained the least (53).

### QTL mapping of ammonia-tolerance

**Fig. 3:**
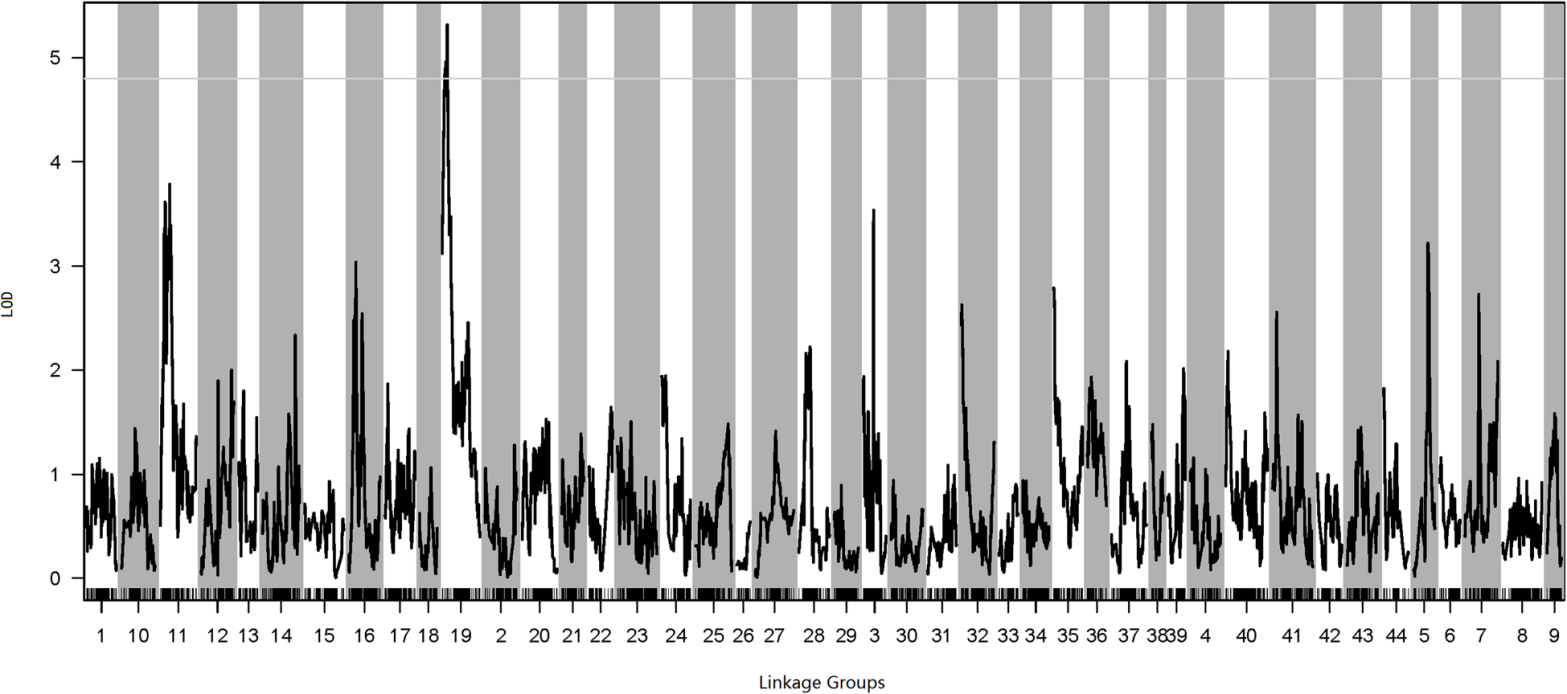
Quantitative trait loci (QTL) for ammonia tolerance in *Litopenaeus vannamei*, showing the logarithm of odds (LOD) values of the linkage groups. The gray line indicates the LOD threshold (4.75; *P* = 0.05)

A QTL analysis of the ammonia-tolerance trait in the LV-N *L. vannamei* family was performed based on the genetic maps. The LOD threshold was 4.75 (1000 permutations, *P* < 0.05). Thus, QTLs with LOD scores > 4.75 were considered effective QTLs. Using this criterion, we identified a QTL within LG19 for ammonia tolerance (Fig. 3). The phenotypic variation explained by this QTL was 7.41–8.46%, the LOD score was 4.75–5.45, and the confidence interval was 12.42–29.43 cM.

### Transcriptome sequencing, candidate gene identification and qRT-PCR verification

The transcriptomes of the 20 most ammonia-tolerant and the 20 most ammonia-sensitive shrimp in each of 4 *L. vannamei* families (LV-N, LV-A, LV-C, and LV-F) with various genetic backgrounds were sequenced. Transcriptome sequencing generated 56.79 Gb of clean data. A total of 7546 DEGs were identified between the ammonia-tolerant and ammonia-sensitive shrimp across all four families: 1869 in LV-A, 2005 in LV-C, 1875 in LV-F, and 1797 in LV-N (Supplementary Material, Table S6).

**Fig. 4:**
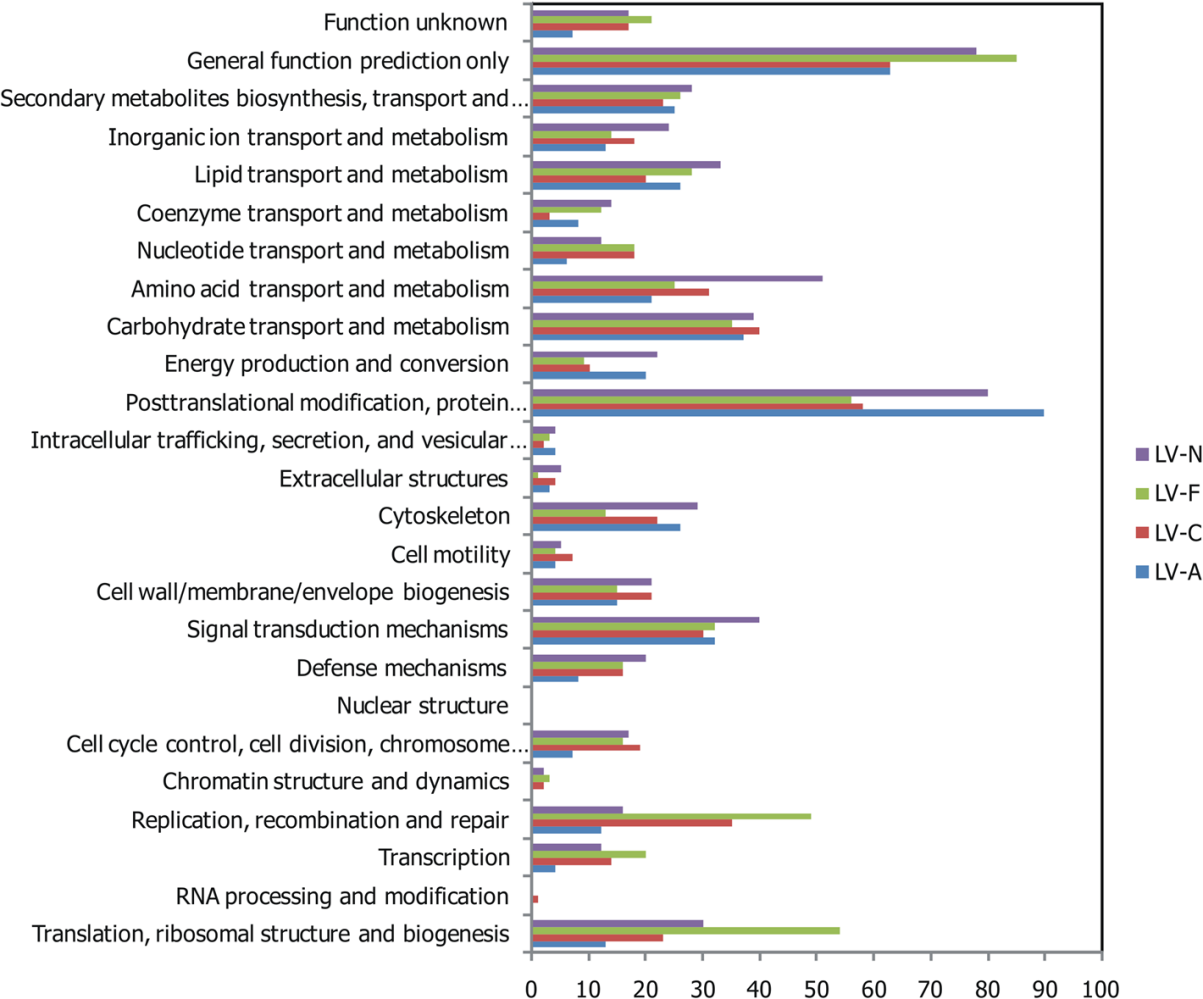
Clusters of Orthologous Groups (COG) classifications of the putative functions of the differentially expressed genes between the most ammonia-tolerant and the most ammonia-sensitive individuals across four families of *Litopenaeus vannamei* (LV-A, LV-C, LV-F, and LV-N)

**Fig. 5:**
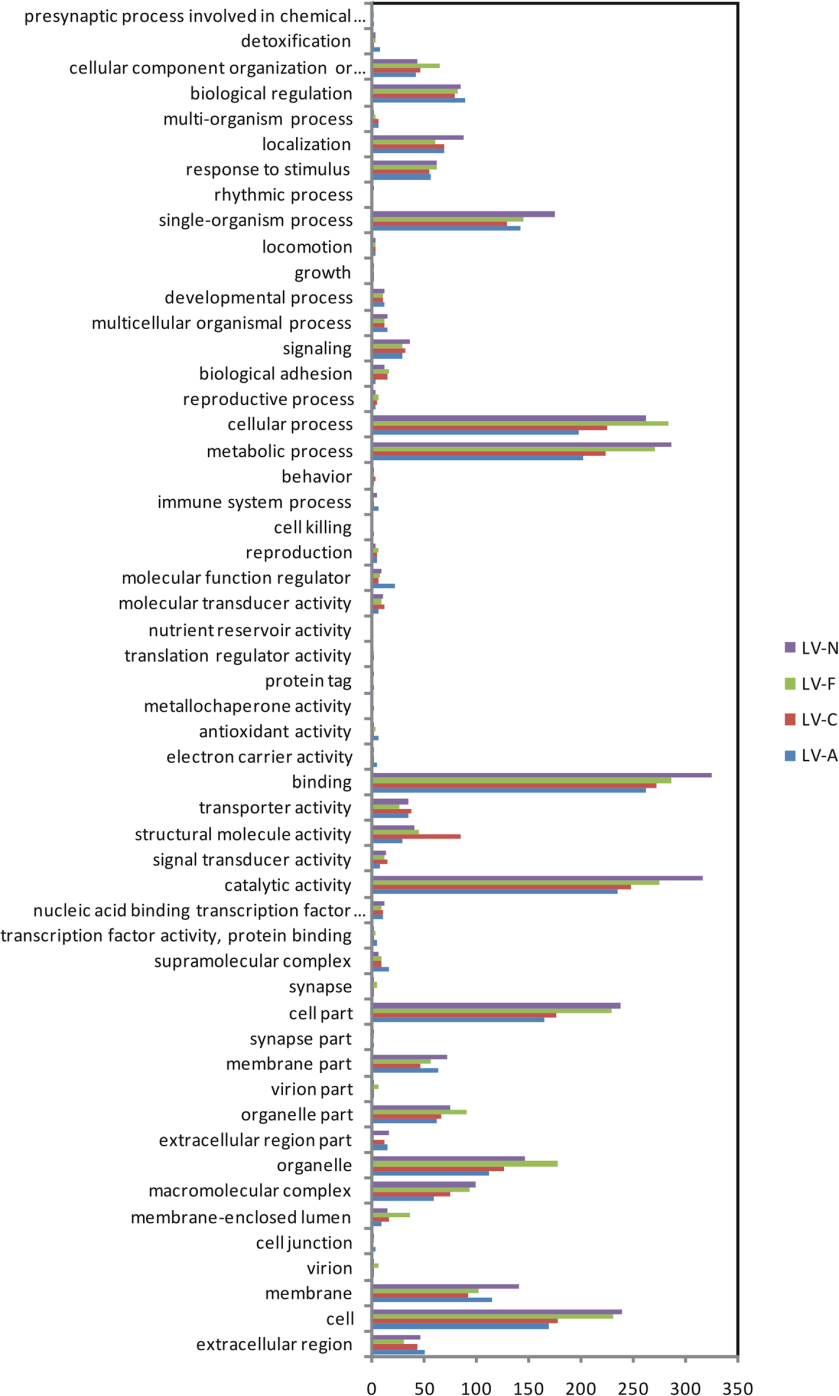
Gene Ontology (GO) classifications of the putative functions of the differentially expressed genes between the most ammonia-tolerant and the most ammonia-sensitive individuals across four families of *Litopenaeus vannamei* (LV-A, LV-C, LV-F, and LV-N)

The numbers of DEG annotations recovered in the databases searched were similar across the 4 *L. vannamei* families. For instance, the COG terms mainly enriched in the DEGs from all four families were posttranslational modification, protein turnover, chaperones, and general function prediction only (Fig. 4); the GO terms primarily enriched in the DEGs from all four families were binding, catalytic activity, cellular process, metabolic process, cell, cell part, single-organism process and membrane functions (Fig. 5).

**Fig. 6:**
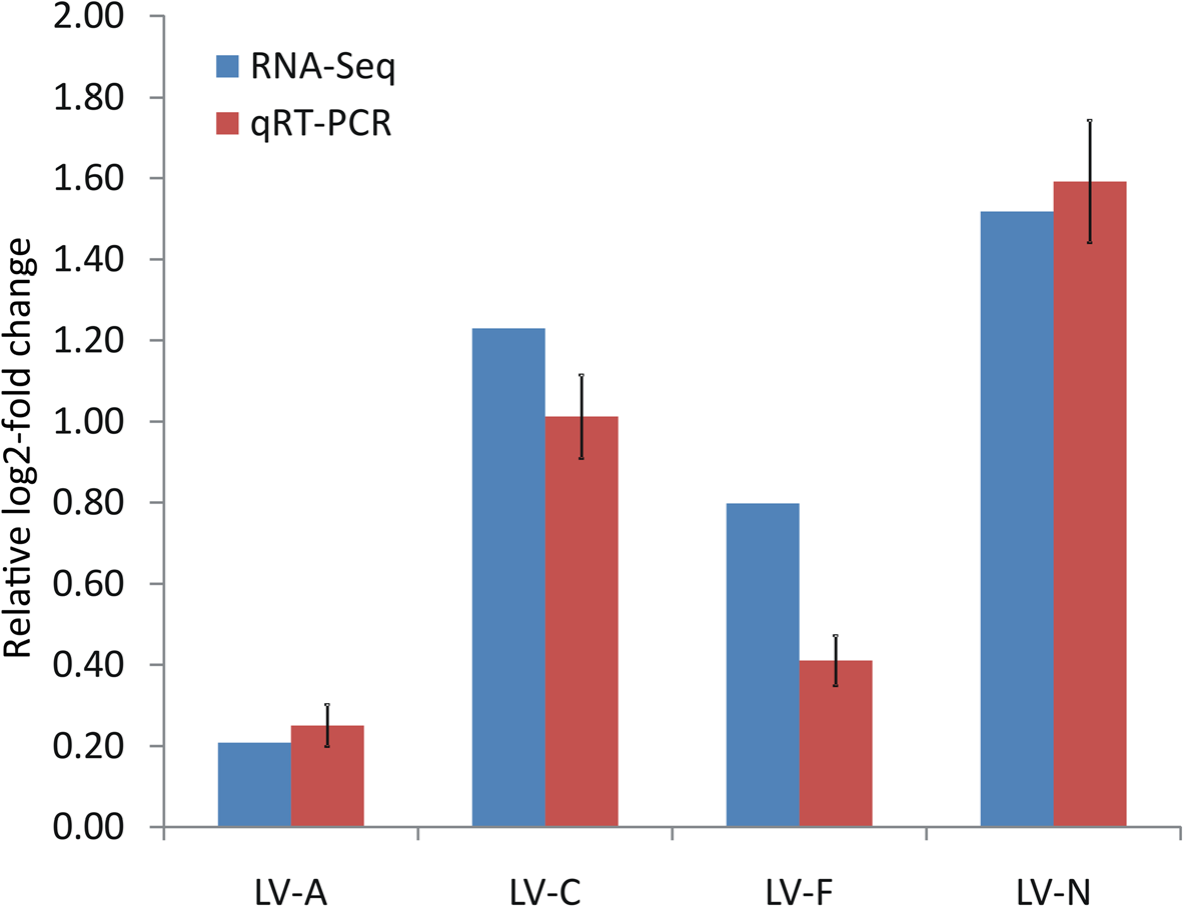
Expression of LOC113809108 gene from the transcriptomic analysis validated by qRT-PCR. Expression of LOC113809108 gene was detected in the most ammonia-tolerant and the most ammonia-sensitive individuals from four families of *Litopenaeus vannamei* (LV-A, LV-C, LV-F, and LV-N). Data were normalized to 18 s rRNA as the reference and presented as a relative log2-fold change to validate the transcriptomic analysis results. Error bars show the standard deviation of three technical replicates

By aligning the DEGs with the QTL region in LG19, 107 DEGs located in the QTL interval were identified. The expression levels and annotations of these DEGs are listed in Supplementary Material Table S7. Of these DEGs, only one gene (LOC113809108) met the criterion used to determine candidate genes associated with ammonia tolerance. This gene was annotated as an ATP synthase g subunit. LOC113809108 was located in the QTL interval, and was significantly upregulated in the most ammonia-tolerant shrimp compared to the most ammonia-sensitive shrimp from families LV-N and LV-C (Fig. 6). This gene was also upregulated in the most ammonia-tolerant shrimp from families LV-A and LV-F, but this difference in expression was not significant (Table 2).

**Table 2:** RNA-Seq analysis showing expression of gene LOC113809108 in the most ammonia-tolerant and the most ammonia-sensitive individuals across four families of *Litopenaeus vannamei* (LV-A, LV-C, LV-F, and LV-N)

Family	Gene LOC113809108 expression level							
	Sensitive group (FPKM)	Sensitive group (count)	Tolerant group (FPKM)	Tolerant group (count)	FDR	Log2-fold Change	Regulated	Difference
LV-A	112.8410	3239	130.2663	2561	0.9589	0.2072	up	Normal
LV-C	63.2980	1651	148.3678	2444	0.0002	1.2289	up	Significant
LV-F	70.4520	1378	122.4527	2258	0.9402	0.7975	up	Normal
LV-N	114.9725	2659	329.0251	8025	0.0001	1.5169	up	Significant

The qRT-PCR analysis showed that the patterns of LOC113809108 gene expression in ammonia-tolerant and ammonia-sensitive pooled samples from the families LV-A, LV-C, LV-F, and LV-N were similar to the patterns determined using RNA-seq: LOC113809108 gene expression was upregulated in the ammonia-tolerant shrimp as compared to the ammonia-sensitive shrimp across all four families (Fig. 6).

## Discussion

This study was aimed at investigating the ammonia tolerance in *L.vannamei* by QTL analysis. A high-density genetic map of *L. vannamei* was constructed using SLAF-seq, and a QTL associated with ammonia tolerance was identified as well as a putative candidate gene associated with ammonia tolerance.

The genome of *L. vannamei* is large (~ 2.45 Gb) [32]. Whole-genome deep resequencing is relatively costly for large genomes and is often not necessary for gene/QTL mapping [43, 44]. In recent years, several genetic linkage maps based on SNPs were constructed and QTL analyses were conducted in *L. vannamei* [30, 45–47]. Yu et al. constructed a high-density genetic map for *L. vannamei* and detected several QTLs for body weight and body length [30]. Yang et al. mapped the sex determination region in *L. vannamei* based on the data used for high-density linkage map construction [45]. Du et al.mapped a QTL for *L. vannamei* gender using a gene-based SNP linkage map [46]. In this study, a high-density genetic map of *L. vannamei* was constructed using SLAF-seq, which is an effective method for discovering large numbers of SNPs and to perform large-scale genotyping [20]. Compared to traditional methods of genetic map construction (e.g., random amplified polymorphic DNA (RAPD), amplified fragment length polymorphism (AFLP), and simple sequence repeat (SSR)), the SLAF-seq method has several advantages for large-scale SNP discovery and genotyping: high density, high throughput, high efficiency, and low cost [48]. Previous studies have developed genetic maps of *L. vannamei* using RAPD, AFLP, and SSR, but in these maps, the average distance between adjacent markers was 1–5 cM [49–52]. The average distance between adjacent markers in the SLAF-seq genetic maps of *L. vannamei* in this study was substantially shorter (0.34 cM). Notably, the average distance between adjacent markers found here was also less than in a previously reported SLAF-seq genetic map of *L. vannamei* (0.75 cM) [30], possibly because a larger sample size and a greater sequencing depth were used. However, the number of LGs in the genetic map of *L. vannamei* in this study (44) was consistent with the number of LGs in the previously reported genetic map of *L. vannamei* [30]. This indicated that *L. vannamei* had 44 chromosomes, which was first reported by CamposRamos [53]. As far as we know, this is the highest density genetic linkage map *L. vannamei*, and it is very useful for comparative analysis of genomic synteny, QTL mapping, positioning of candidate genes, and marker-assisted selection.

As most animals cannot self-fertilize, it is difficult to develop common populations for genetic mapping (e.g., F2, recombinant inbred line (RIL), and nearlyisogenic line (NIL) populations). Therefore, an F1 population of *L. vannamei* was used to construct the genetic map, relying on a pseudo-testcross strategy. This strategy was based on the selection of single-dose markers present in one parent and absent in the other, and carried at a 1:1 ratio by the F1 offspring [54]. Therefore, gamete separation in each individual can be directly analyzed. The pseudo-testcross strategy has been widely used to construct animal F1 populations for genetic mapping [55–58]. In this study, an F1 population was developed using one ammonia-tolerant male parent (the result of 10 generations of selective breeding) and one female shrimp from a common family.

Previous studies have suggested that the size of the mapped population might affect the accuracy of the genetic map and the QTL analysis, and have shown that genetic map accuracy increases with the size of the population used [59]. Specifically, populations of > 200 individuals are considered sufficient for the construction of accurate genetic maps [59]. Thus, 284 randomly-selected individuals from the F1 population were used to construct the linkage map. However, the determination of shrimp survival time during the ammonia stress experiment depended on human observation, and thus may not have been perfectly accurate. To reduce the possible impacts of measurement inaccuracies on the QTL analysis, a relatively large F1 population was used. This larger population increased the accuracy of the QTL mapping, compensating for any instances of human error in survival time measurement.

Ammonia stress is one of the biggest challenges facing shrimp aquaculture. Ammonia not only has a direct lethal effect on shrimp [7], but also inhibits the shrimp immune system and increases sensitivity to pathogens [60]. Breeding new varieties of ammonia-tolerant shrimp is therefore an important target of the shrimp aquaculture industry. Some researches on shrimp ammonia tolerance have been carried out in recent years. Lu et al. found 12 SNPs associated with ammonia tolerance in *L. vannamei* using marker-trait correlation analyses, and these SNPs were identified in the thrombospondin gene and X-box binding protein 1 gene [15]. At the same time, Lu et al. identified 202 differentially expressed proteins (DEPs) between ammonia-tolerant and ammonia-sensitive *L. vannamei* using a comparative proteome analysis based on iTRAQ technique, and 77.8% of the DEPs were reported mainly involving in immune defense and stress tolerant in crustacean species [16]. Here, a QTL for ammonia-tolerance, located on LG19 at 169.09–169.49 cM was identified, that explained 7.41–8.46% of the phenotypic variation in ammonia tolerance. To the best of our knowledge, this is the first QTL for ammonia-tolerance reported in shrimp. However, having one QTL that explains about 8% of the variance indeed is not enough to initiate genetic breeding. Therefore in future research, the cutoff of LOD score can be relaxed to get more QTLs, intervals, and candidate DEGs. And then, more candidate genes related to ammonia tolerance should be identified. Moreover, independent families should be used to verify whether the QTL is common across different populations.

A QTL usually spans a large chromosomal region and may contain hundreds of genes. Therefore, in order to identify functional genes associated with ammonia tolerance, the transcriptomes of 4 *L. vannamei* families (LV-N, LV-A, LV-C, and LV-F) with different genetic backgrounds were sequenced. Combining QTL mapping and gene expression analysis, we identified a single DEG (LOC113809108), located in the QTL interval, that was annotated as an ATP synthase g subunit and was significantly upregulated in the ammonia-tolerant LV-N and LV-C shrimp. The ATP synthase g subunit is located in F0 portion of ATP synthase, which consists of a membrane-integrated portion (F0 complex) and a membrane-protruding portion (F1 complex) [61]. The ATP synthase g subunit is essential for the formation of the F0 complex [61]. In the previous studies [15, 16], ATP synthase has not been identified as being related to shrimp ammonia tolerance. ATP synthase is a double-motor enzyme that is involved in ATP synthesis, ATP hydrolysis-dependent processes, and the regulation of the proton gradient across some membrane-dependent systems [62]. Several studies have shown that ammonia excretion in aquatic animals is associated with Na^+^/K^+^-ATPase, which is mainly located on the basolateral membrane of branchial cells; NH_4_^+^ is excreted into the environment when K^+^ is replaced by NH_4_^+^ via the Na^+^/NH_4_^+^ exchanger [63–65]. Indeed, a previous study suggested that in *L. vannamei*, high ammonia tolerance was mainly a result of improved ammonia excretion and detoxification, as well as an accelerated energy metabolism [17]. Therefore, we speculate that ATP synthesis might affect the ammonia tolerance of *L. vannamei* by regulating ATP synthesis and controlling cellular ammonia excretion. The results would provide useful information for further study of the molecular mechanisms of ammonia adaptive strategies in shrimps.

## Conclusions

In this study, we constructed a high-density genetic map of *L. vannamei* and identified a QTL for ammonia tolerance. By combining QTL and transcriptome analyses, we identified a candidate gene associated with ammonia tolerance. The results help us better understand the molecular mechanism of ammonia tolerance in shrimp. Our work provides the basis for future genetic studies focused on molecular marker-assisted selective breeding.

## Supplementary Information

**Additional file 1: Table S1.** Median lethal concentration of NH4Cl for *Litopenaeus vannamei* families LV-A, LV-C, LV-F, and LV-N.

**Additional file 2: Table S2.** Summary of the SLAF sequencing of *Oryza sativa* japonica.

**Additional file 3: Table S3.** Basic information of the male map.

**Additional file 4: Table S4.** Basic information of the female map.

**Additional file 5: Table S5.** Basic information of the sex-average map

**Additional file 6: Table S6.** Statistics of the number of differentially expressed genes (DEGs) between the most ammonia-sensitive and most ammonia-tolerant shrimp in the four experimental families.

**Additional file 7: Table S7.** DEGs within the QTL interval and their expression levels and annotations

**Additional file 8: Figure S1.** The accumulated mortality rate of shrimp in LV-N family under an acute ammonia stress (ammonia-N concentration of 345.94 mg/L, temperature of 27.0 ± 0.5 °C, pH of 8.1 ± 0.2, salinity of 30.2‰, and dissolved oxygen of 6–8 mg/L).

## Data Availability

All data generated or analysed during this study are included in this published article and its supplementary information files. Raw SLAF sequencing reads are deposited in NCBI database under the accession numbers PRJNA545592. Raw RNA-seq are deposited in NCBI database under the accession numbers SRR9822091, SRR9822090, SRR9822095, SRR9822094, SRR9822098, SRR9822099, SRR9822093, and SRR9822085.

## Abbreviations

cM: Centimorgans
SLAF-seq: Specific length amplified fragment sequencing
QTL: Quantitative trait locus
ammonia-N: Ammonia nitrogen
SNPs: Single nucleotide polymorphisms
PCRs: Polymerase chain reactions
LOD: Logarithm of odds
qRT-PCR: Quantitative real-time PCR
FPKM: Fragments per kilobase of transcript per million mapped reads
FDR: False discovery rate
DEGs: Differentially expressed genes
Gb: Gigabases
Mb: Megabases
LGs: Linkage groups
