# Supplementary material for: Identification of a quantitative trait loci (QTL) associated with ammonia tolerance in the Pacific white shrimp (Litopenaeus vannamei)
Source: BMC Genomics. 2020 Dec 2;21:857. doi: 10.1186/s12864-020-07254-x (PMC7709431; doi:10.1186/s12864-020-07254-x)
Supplement: Supplementary file 1 — Additional file 1: Table S1. Median lethal concentration of NH4Cl for Litopenaeus vannamei families LV-A, LV-C, LV-F, and LV-N. [file 12864_2020_7254_MOESM1_ESM.docx]

**Supplementary Table S1.** Median lethal concentration (LC50) of NH_4_Cl for *Litopenaeus vannamei* families LV-A, LV-C, LV-F, and LV-N.

| Family | 24h-LC50（mg/L） | 48h-LC50（mg/L） | 72h-LC50（mg/L） | 96h-LC50（mg/L） |
| --- | --- | --- | --- | --- |
| LV-A | 140.96 | 104.34 | 84.61 | 70.65 |
| LV-C | 189.19 | 104.36 | 77.93 | 71.93 |
| LV-F | 117.88 | 82.43 | 70.60 | 70.60 |
| LV-N | 137.26 | 84.44 | 71.24 | 67.33 |

Note: pH maintained at 8.2 ± 0.3; temperature maintained at 27.0 ± 0.5°C; salinity maintained at 30.1‰; and dissolved oxygen maintained at 7–8 mg/L.
