# Supplementary material for: Identification of a quantitative trait loci (QTL) associated with ammonia tolerance in the Pacific white shrimp (Litopenaeus vannamei)
Source: BMC Genomics. 2020 Dec 2;21:857. doi: 10.1186/s12864-020-07254-x (PMC7709431; doi:10.1186/s12864-020-07254-x)
Supplement: Supplementary file 2 — Additional file 2: Table S2. Summary of the SLAF sequencing of Oryza sativa japonica. [file 12864_2020_7254_MOESM2_ESM.docx]

| **Table S2. Summary of the SLAF sequencing of *Oryza sativa japonica*** | |
| --- | --- |
| Information of the map | Value |
| Total bases | 343.21 Mb |
| Total reads | 1.72 Mb |
| Average Q30 | 95.81% |
| Average GC | 40.96% |
| Enzyme digestion protocol | HaeIII+Hpy166II |
| Restriction fragment length | 314-414 bp |
| Paired-end mapped reads | 91.43% |
| Enzymatic digestion efficiency | 92.19% |
