# Supplementary material for: Identification of a quantitative trait loci (QTL) associated with ammonia tolerance in the Pacific white shrimp (Litopenaeus vannamei)
Source: BMC Genomics. 2020 Dec 2;21:857. doi: 10.1186/s12864-020-07254-x (PMC7709431; doi:10.1186/s12864-020-07254-x)
Supplement: Supplementary file 3 — Additional file 3: Table S3. Basic information of the male map. [file 12864_2020_7254_MOESM3_ESM.docx]

| **Table S3. Basic information of the male map** | | |  |  |  |
| --- | --- | --- | --- | --- | --- |
| **Linkage** | **Total** | **Total** | **Average** | **Max** | **Gap** |
| **Group ID** | **Marker** | **Distance(cM)** | **Distance(cM)** | **Gap (cM)** | **< 5 cM（%）** |
| 1 | 310 | 135.26 | 0.44 | 12.77 | 98.06 |
| 2 | 234 | 176.34 | 0.75 | 26.76 | 97.85 |
| 3 | 160 | 93.81 | 0.59 | 14.69 | 98.74 |
| 4 | 264 | 139.31 | 0.53 | 20.1 | 96.2 |
| 5 | 177 | 79.92 | 0.45 | 15.57 | 98.3 |
| 6 | 219 | 115.78 | 0.53 | 18.04 | 98.62 |
| 7 | 111 | 193.9 | 1.75 | 32.07 | 89.09 |
| 8 | 379 | 242.8 | 0.64 | 10.46 | 98.41 |
| 9 | 134 | 53.92 | 0.4 | 3.27 | 100 |
| 10 | 169 | 167.12 | 0.99 | 21.7 | 93.45 |
| 11 | 290 | 177.14 | 0.61 | 64.61 | 98.27 |
| 12 | 462 | 240.42 | 0.52 | 50.23 | 98.05 |
| 13 | 197 | 90.98 | 0.46 | 15.09 | 97.96 |
| 14 | 452 | 168.05 | 0.37 | 24.54 | 98.89 |
| 15 | 413 | 206.02 | 0.5 | 27.8 | 98.3 |
| 16 | 357 | 191.25 | 0.54 | 7.59 | 99.16 |
| 17 | 75 | 100.7 | 1.34 | 13.19 | 90.54 |
| 18 | 252 | 79.43 | 0.32 | 13.22 | 99.2 |
| 19 | 274 | 227.63 | 0.83 | 52.79 | 97.8 |
| 20 | 341 | 187.35 | 0.55 | 13.63 | 98.24 |
| 21 | 306 | 103.5 | 0.34 | 7.85 | 98.69 |
| 22 | 154 | 108.4 | 0.7 | 25.07 | 97.39 |
| 23 | 296 | 211.85 | 0.72 | 17.53 | 97.97 |
| 24 | 231 | 114.01 | 0.49 | 8 | 97.39 |
| 25 | 352 | 204.66 | 0.58 | 22.42 | 98.29 |
| 26 | 39 | 78.08 | 2 | 9.4 | 84.21 |
| 27 | 151 | 264.77 | 1.75 | 26.74 | 90 |
| 28 | 183 | 147.42 | 0.81 | 40.63 | 96.7 |
| 29 | 280 | 150.45 | 0.54 | 6.78 | 98.57 |
| 30 | 487 | 198.13 | 0.41 | 54.23 | 98.97 |
| 31 | 585 | 173.32 | 0.3 | 72.97 | 99.14 |
| 32 | 332 | 188.66 | 0.57 | 33.96 | 99.09 |
| 33 | 225 | 58.53 | 0.26 | 5.19 | 99.55 |
| 34 | 305 | 196.63 | 0.64 | 9.12 | 99.34 |
| 35 | 273 | 146.65 | 0.54 | 23.92 | 98.16 |
| 36 | 250 | 87.16 | 0.35 | 26.25 | 98.8 |
| 37 | 199 | 127.69 | 0.64 | 39.85 | 98.48 |
| 38 | 135 | 54.27 | 0.4 | 8.84 | 99.25 |
| 39 | 215 | 83.64 | 0.39 | 14.62 | 98.6 |
| 40 | 373 | 239.85 | 0.64 | 22.8 | 98.12 |
| 41 | 242 | 196.26 | 0.81 | 14.63 | 94.19 |
| 42 | 112 | 122.29 | 1.09 | 41.43 | 97.3 |
| 43 | 313 | 155.69 | 0.5 | 28.82 | 98.72 |
| 44 | 204 | 125.95 | 0.62 | 31.92 | 97.04 |
| Total | 11,512 | 6,604.99 | 0.58 | 72.97 | 97.25 |
|  |  |  |  |  |  |
