# Supplementary material for: Identification of a quantitative trait loci (QTL) associated with ammonia tolerance in the Pacific white shrimp (Litopenaeus vannamei)
Source: BMC Genomics. 2020 Dec 2;21:857. doi: 10.1186/s12864-020-07254-x (PMC7709431; doi:10.1186/s12864-020-07254-x)
Supplement: Supplementary file 4 — Additional file 4: Table S4. Basic information of the female map. [file 12864_2020_7254_MOESM4_ESM.docx]

| **Table S4. Basic information of the female map** | | |  |  |  |
| --- | --- | --- | --- | --- | --- |
| **Linkage** | **Total** | **Total** | **Average** | **Max** | **Gap** |
| **Group ID** | **Marker** | **Distance(cM)** | **Distance(cM)** | **Gap (cM)** | **< 5 cM（%）** |
| 1 | 361 | 162.38 | 0.45 | 35.37 | 98.89 |
| 2 | 183 | 138.8 | 0.76 | 58.51 | 99.45 |
| 3 | 183 | 127.89 | 0.7 | 5.56 | 98.35 |
| 4 | 160 | 167 | 1.04 | 49.28 | 94.97 |
| 5 | 156 | 130.79 | 0.84 | 54.23 | 96.13 |
| 6 | 49 | 70 | 1.43 | 51.2 | 95.83 |
| 7 | 323 | 134.99 | 0.42 | 19.14 | 97.83 |
| 8 | 365 | 141.11 | 0.39 | 27.01 | 99.18 |
| 9 | 121 | 78.83 | 0.65 | 20.54 | 98.33 |
| 10 | 163 | 175.43 | 1.08 | 49.28 | 96.91 |
| 11 | 317 | 180.98 | 0.57 | 26.75 | 98.1 |
| 12 | 187 | 83.73 | 0.45 | 12.98 | 96.77 |
| 13 | 234 | 98.99 | 0.42 | 17.53 | 98.71 |
| 14 | 249 | 189.41 | 0.76 | 21.16 | 96.77 |
| 15 | 99 | 82.68 | 0.84 | 17.16 | 95.92 |
| 16 | 182 | 112.7 | 0.62 | 11.42 | 98.9 |
| 17 | 237 | 168.55 | 0.71 | 14.62 | 97.46 |
| 18 | 317 | 84.58 | 0.27 | 16.54 | 98.42 |
| 19 | 244 | 141.46 | 0.58 | 22.09 | 97.94 |
| 20 | 332 | 164.02 | 0.49 | 17.73 | 97.89 |
| 21 | 208 | 67.65 | 0.33 | 7.52 | 99.03 |
| 22 | 150 | 129.15 | 0.86 | 52.19 | 97.32 |
| 23 | 336 | 183.94 | 0.55 | 8.84 | 97.61 |
| 24 | 297 | 177.3 | 0.6 | 86.85 | 98.31 |
| 25 | 346 | 146.62 | 0.42 | 30.6 | 99.13 |
| 26 | 24 | 45.79 | 1.91 | 7.2 | 91.3 |
| 27 | 21 | 45.55 | 2.17 | 27.44 | 95 |
| 28 | 216 | 125.06 | 0.58 | 22.9 | 99.07 |
| 29 | 254 | 79.99 | 0.31 | 6.87 | 99.6 |
| 30 | 357 | 114.14 | 0.32 | 20.1 | 99.16 |
| 31 | 146 | 109.02 | 0.75 | 21.7 | 95.17 |
| 32 | 471 | 141.24 | 0.3 | 14.15 | 98.72 |
| 33 | 249 | 106.75 | 0.43 | 15.91 | 97.18 |
| 34 | 142 | 60.06 | 0.42 | 5.52 | 98.58 |
| 35 | 383 | 142.26 | 0.37 | 58.56 | 99.21 |
| 36 | 540 | 96.14 | 0.18 | 26.25 | 99.44 |
| 37 | 137 | 175.37 | 1.28 | 18.54 | 94.12 |
| 38 | 36 | 28.17 | 0.78 | 8.42 | 97.14 |
| 39 | 256 | 86.7 | 0.34 | 20.1 | 99.22 |
| 40 | 211 | 169 | 0.8 | 20.8 | 95.71 |
| 41 | 380 | 207.96 | 0.55 | 11.42 | 97.36 |
| 42 | 178 | 121.38 | 0.68 | 9.08 | 98.31 |
| 43 | 203 | 156.61 | 0.77 | 17.42 | 96.04 |
| 44 | 290 | 126.03 | 0.43 | 23.36 | 97.92 |
| Total | 10,293 | 5,476.20 | 0.53 | 86.85 | 97.55 |
|  |  |  |  |  |  |
