# Supplementary material for: Identification of a quantitative trait loci (QTL) associated with ammonia tolerance in the Pacific white shrimp (Litopenaeus vannamei)
Source: BMC Genomics. 2020 Dec 2;21:857. doi: 10.1186/s12864-020-07254-x (PMC7709431; doi:10.1186/s12864-020-07254-x)
Supplement: Supplementary file 5 — Additional file 5: Table S5. Basic information of the sex-average map [file 12864_2020_7254_MOESM5_ESM.docx]

| **Table S5. Basic information of the sex-average map** | | | |  |  |
| --- | --- | --- | --- | --- | --- |
| **Linkage** | **Total** | **Total** | **Average** | **Max** | **Gap** |
| **Group ID** | **Marker** | **Distance(cM)** | **Distance(cM)** | **Gap (cM)** | **< 5 cM（%）** |
| 1 | 537 | 150.25 | 0.28 | 8.01 | 99.25 |
| 2 | 341 | 162.2 | 0.48 | 16.76 | 98.53 |
| 3 | 266 | 114.15 | 0.43 | 8.98 | 99.62 |
| 4 | 345 | 153.87 | 0.45 | 19.19 | 96.8 |
| 5 | 245 | 107.34 | 0.44 | 13.73 | 97.95 |
| 6 | 251 | 101.81 | 0.41 | 10.34 | 98.4 |
| 7 | 343 | 164.44 | 0.48 | 12.07 | 97.95 |
| 8 | 514 | 198.53 | 0.39 | 18.74 | 99.61 |
| 9 | 209 | 74.13 | 0.36 | 15.51 | 99.52 |
| 10 | 249 | 171.28 | 0.69 | 13.89 | 95.97 |
| 11 | 444 | 180.67 | 0.41 | 10.49 | 98.42 |
| 12 | 553 | 165.37 | 0.3 | 17.57 | 98.73 |
| 13 | 348 | 94.99 | 0.27 | 9.56 | 99.42 |
| 14 | 557 | 186.69 | 0.34 | 9.36 | 98.92 |
| 15 | 477 | 197.66 | 0.42 | 19.89 | 98.53 |
| 16 | 413 | 157.55 | 0.38 | 7.79 | 99.76 |
| 17 | 280 | 149.76 | 0.54 | 9.83 | 98.57 |
| 18 | 407 | 88.03 | 0.22 | 6.61 | 99.01 |
| 19 | 420 | 190.35 | 0.45 | 12.33 | 97.85 |
| 20 | 492 | 175.86 | 0.36 | 11.02 | 98.57 |
| 21 | 398 | 110.62 | 0.28 | 7.85 | 98.99 |
| 22 | 247 | 119.66 | 0.49 | 16.03 | 98.37 |
| 23 | 372 | 197.9 | 0.53 | 11.45 | 99.19 |
| 24 | 431 | 146.01 | 0.34 | 16.28 | 98.84 |
| 25 | 511 | 182.19 | 0.36 | 17.72 | 99.22 |
| 26 | 53 | 67.84 | 1.3 | 5.6 | 96.15 |
| 27 | 166 | 195.29 | 1.18 | 16 | 93.94 |
| 28 | 340 | 153.75 | 0.45 | 16.79 | 98.82 |
| 29 | 415 | 120.95 | 0.29 | 5.84 | 99.76 |
| 30 | 632 | 160.59 | 0.25 | 10.7 | 98.89 |
| 31 | 695 | 144.34 | 0.21 | 15.83 | 99.28 |
| 32 | 595 | 165.31 | 0.28 | 16.15 | 99.49 |
| 33 | 399 | 95.12 | 0.24 | 8.14 | 98.74 |
| 34 | 371 | 130.53 | 0.35 | 5.12 | 99.73 |
| 35 | 494 | 144.82 | 0.29 | 12.5 | 98.99 |
| 36 | 686 | 95.4 | 0.14 | 14.29 | 99.71 |
| 37 | 318 | 179 | 0.56 | 8.99 | 98.11 |
| 38 | 147 | 56.3 | 0.39 | 4.92 | 100 |
| 39 | 370 | 87.35 | 0.24 | 13.18 | 99.73 |
| 40 | 492 | 210.74 | 0.43 | 12.19 | 98.57 |
| 41 | 510 | 202.11 | 0.4 | 11.69 | 98.62 |
| 42 | 237 | 122.37 | 0.52 | 13.05 | 97.88 |
| 43 | 395 | 160.12 | 0.41 | 15.8 | 98.48 |
| 44 | 373 | 126.88 | 0.34 | 17.12 | 98.39 |
| Total | 17,338 | 6,360.12 | 0.37 | 19.89 | 98.62 |
|  |  |  |  |  |  |
