# Supplementary material for: Identification of a quantitative trait loci (QTL) associated with ammonia tolerance in the Pacific white shrimp (Litopenaeus vannamei)
Source: BMC Genomics. 2020 Dec 2;21:857. doi: 10.1186/s12864-020-07254-x (PMC7709431; doi:10.1186/s12864-020-07254-x)
Supplement: Supplementary file 6 — Additional file 6: Table S6. Statistics of the number of differentially expressed genes (DEGs) between the most ammonia-sensitive and most ammonia-tolerant shrimp in the four experimental families. [file 12864_2020_7254_MOESM6_ESM.docx]

| **Table S6. Statistics of the number of differentially expressed genes (DEGs) between the most ammonia-sensitive and most ammonia-tolerant shrimp in the four experimental families.** | | | |
| --- | --- | --- | --- |
| Family | DEG Number | up-regulated | down-regulated |
| LV-A | 1,869 | 597 | 1,272 |
| LV-C | 2,005 | 1,431 | 574 |
| LV-F | 1,875 | 1,391 | 484 |
| LV-N | 1,797 | 821 | 976 |
|  |  |  |  |
