# Supplementary figures and images for: Identification of a quantitative trait loci (QTL) associated with ammonia tolerance in the Pacific white shrimp (Litopenaeus vannamei)
Source: BMC Genomics. 2020 Dec 2;21:857. doi: 10.1186/s12864-020-07254-x (PMC7709431; doi:10.1186/s12864-020-07254-x)

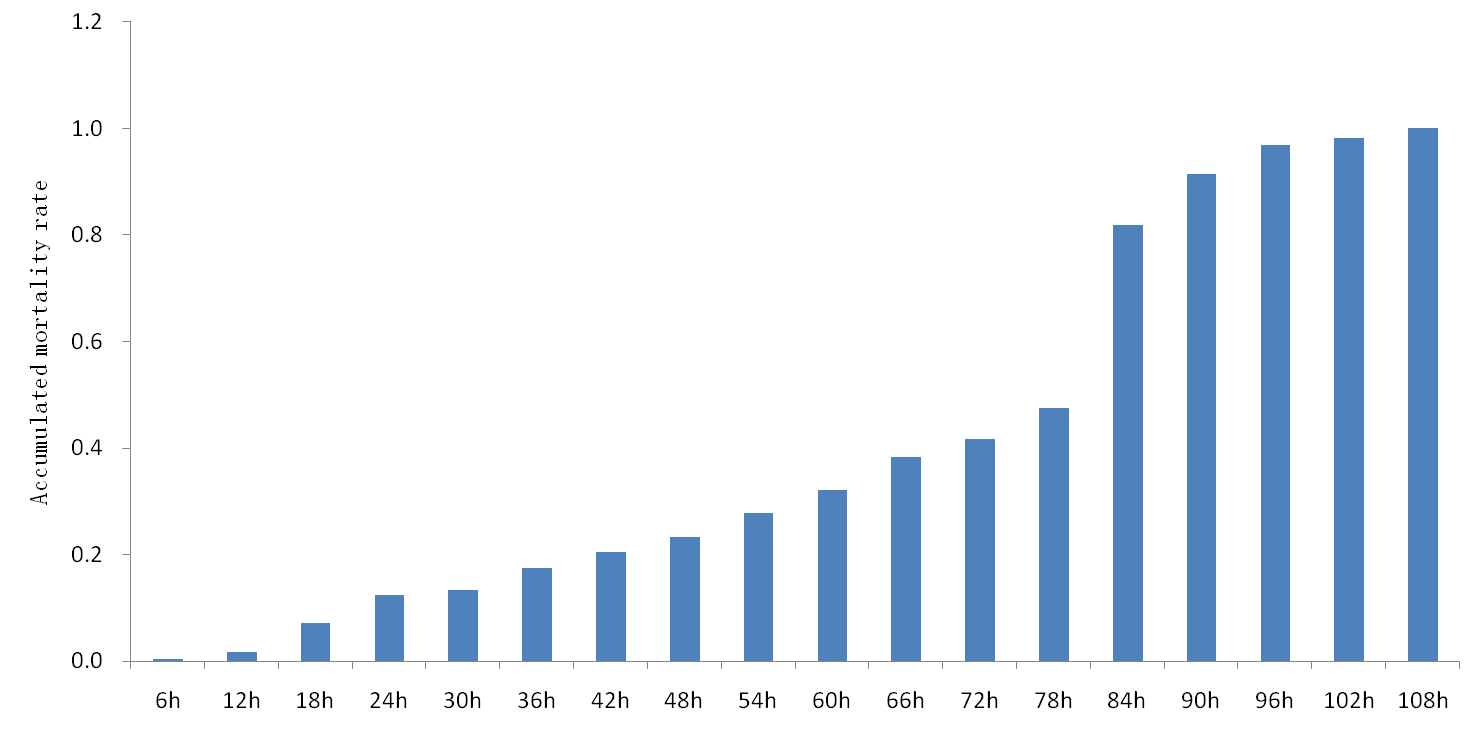

Supplement: Supplementary file 8 — Additional file 8: Figure S1. The accumulated mortality rate of shrimp in LV-N family under an acute ammonia stress (ammonia-N concentration of 345.94 mg/L, temperature of 27.0 ± 0.5 °C, pH of 8.1 ± 0.2, salinity of 30.2‰, and dissolved oxygen of 6–8 mg/L). [file 12864_2020_7254_MOESM8_ESM.tif]
